# Supplementary material for: Biological Activity and In Silico Study of 3-Modified Derivatives of Betulin and Betulinic Aldehyde
Source: Int J Mol Sci. 2019 Mar 19;20(6):1372. doi: 10.3390/ijms20061372 (PMC6471197; doi:10.3390/ijms20061372)
Supplement: Supplementary file 1 [file ijms-20-01372-s001.pdf]

Supplementary material

^{1}H NMR and ^{13}C NMR spectra of 3-modified betulin derivatives **5-14**

Figure S1: ^{1}H NMR spectrum of compound **5**

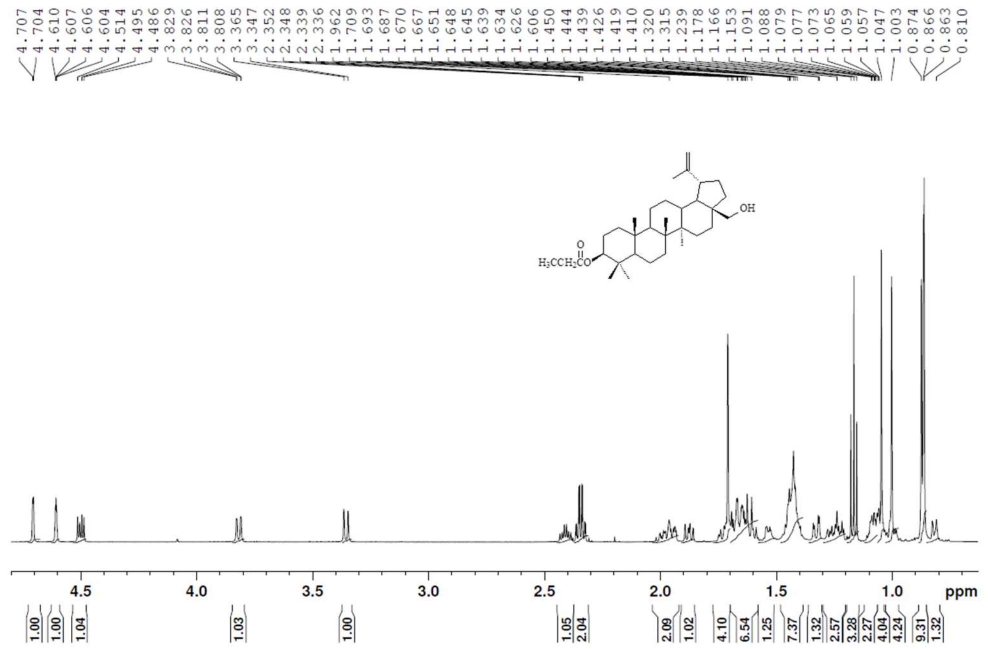

Figure S2: ^{13}C NMR spectrum of compound **5**

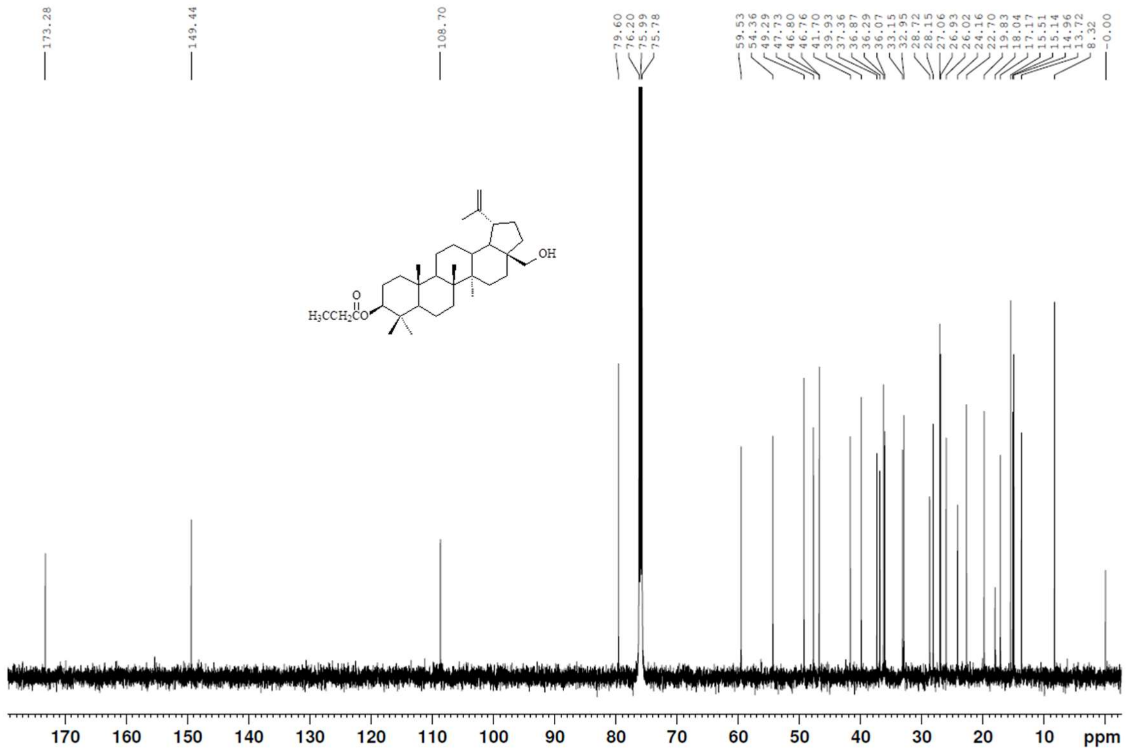

Figure S3: ^{1}H NMR spectrum of compound **6**

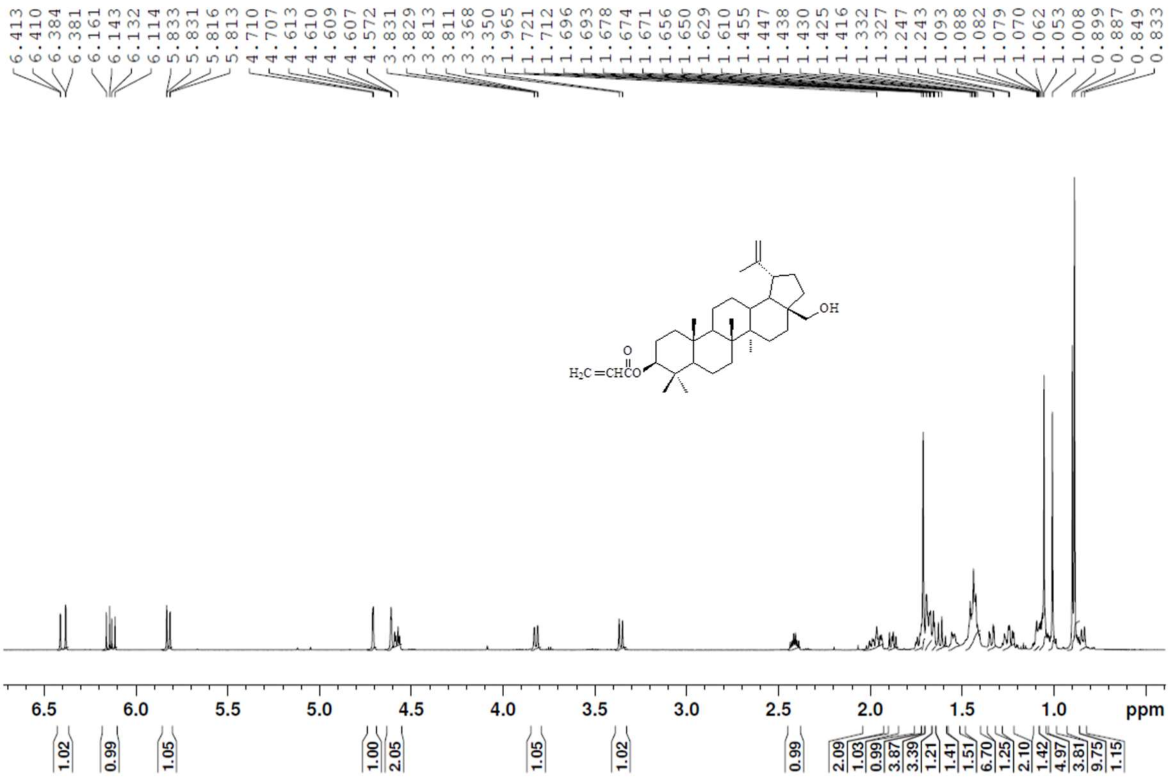

Figure S4: ^{13}C NMR spectrum of compound **6**

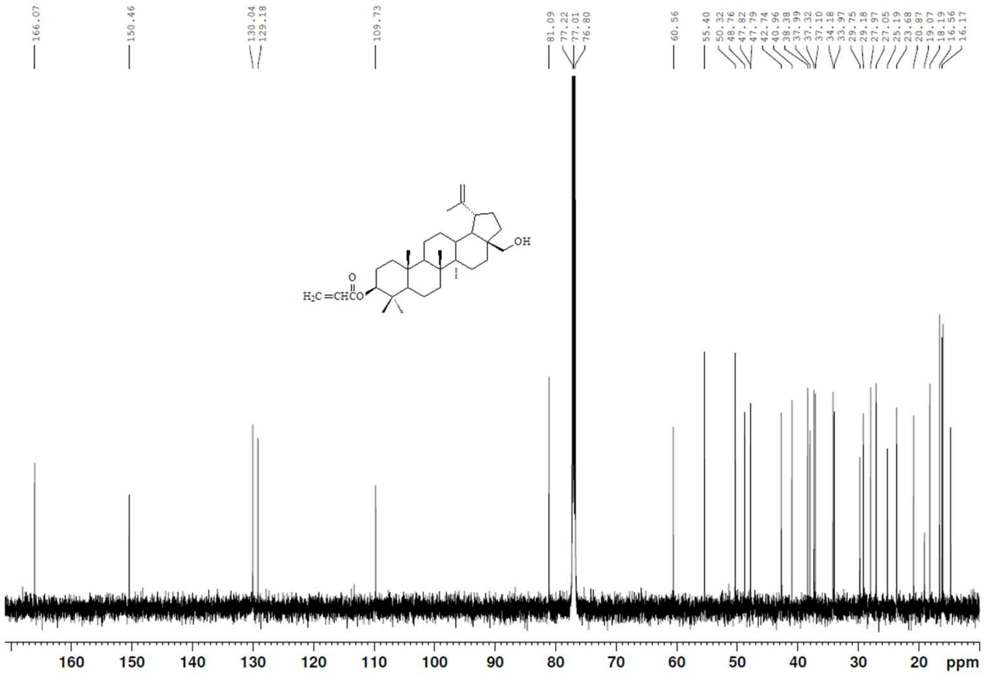

Figure S5: ^{1}H NMR spectrum of compound **7**

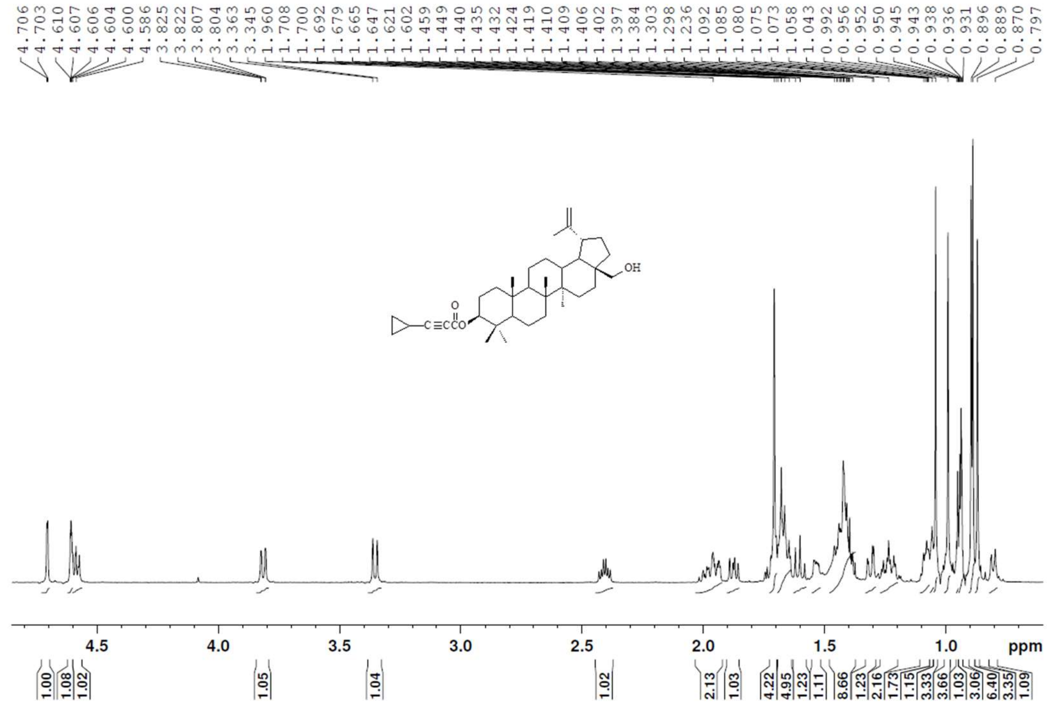

Figure S6: ^{13}C NMR spectrum of compound **7**

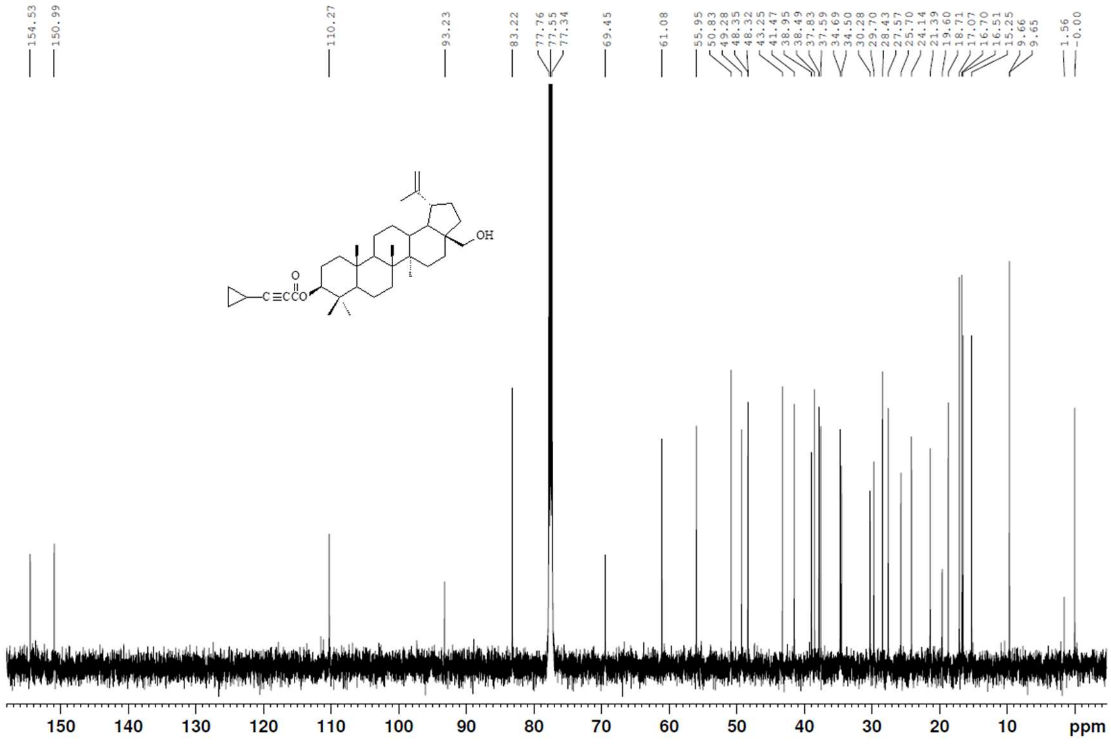

Figure S7: ^{1}H NMR spectrum of compound **8**

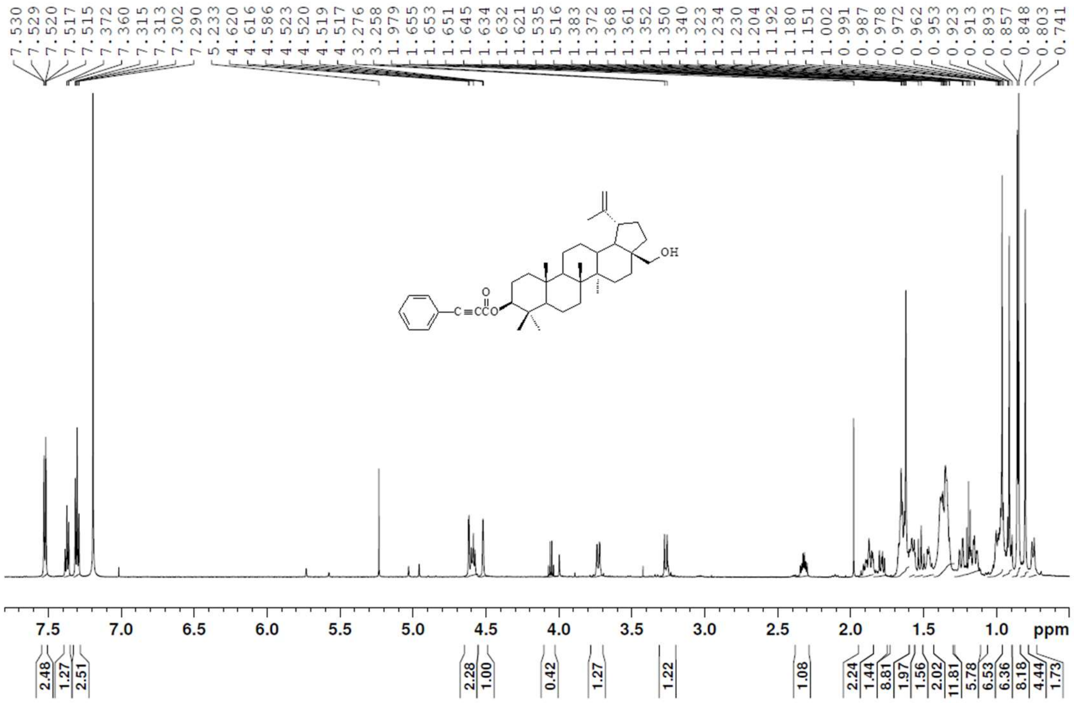

Figure S8: ^{13}C NMR spectrum of compound **8**

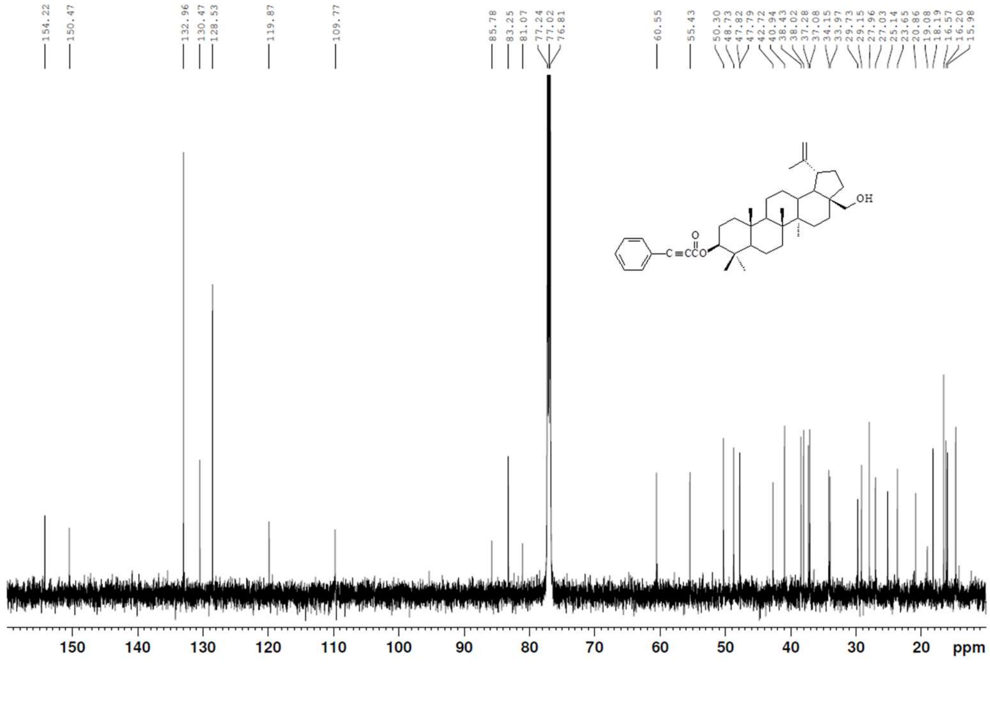

Figure S9: ^{1}H NMR spectrum of compound **9**

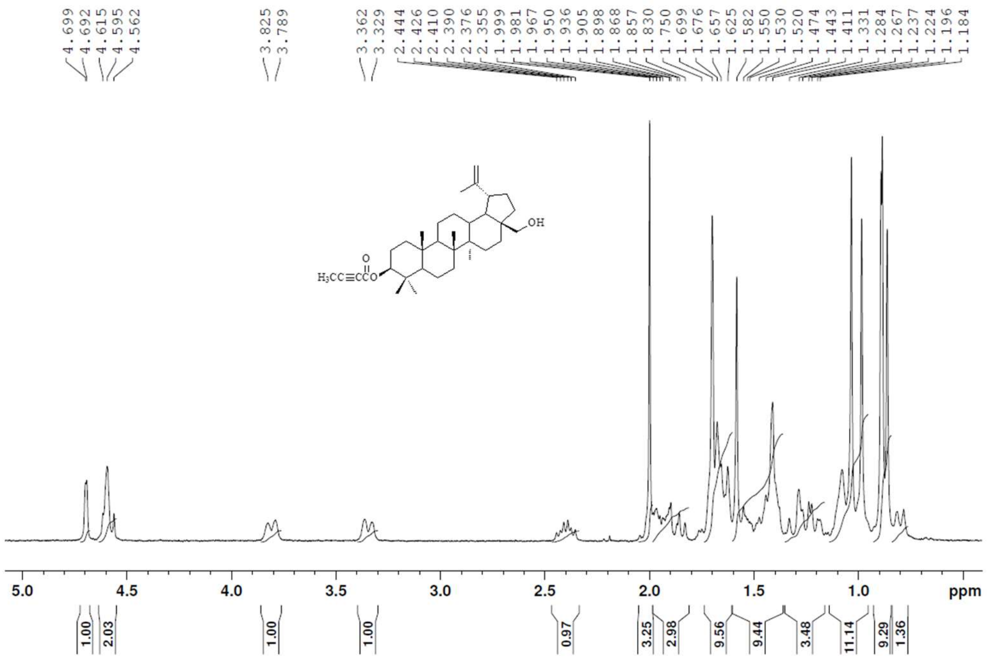

Figure S10: ^{13}C NMR spectrum of compound **9**

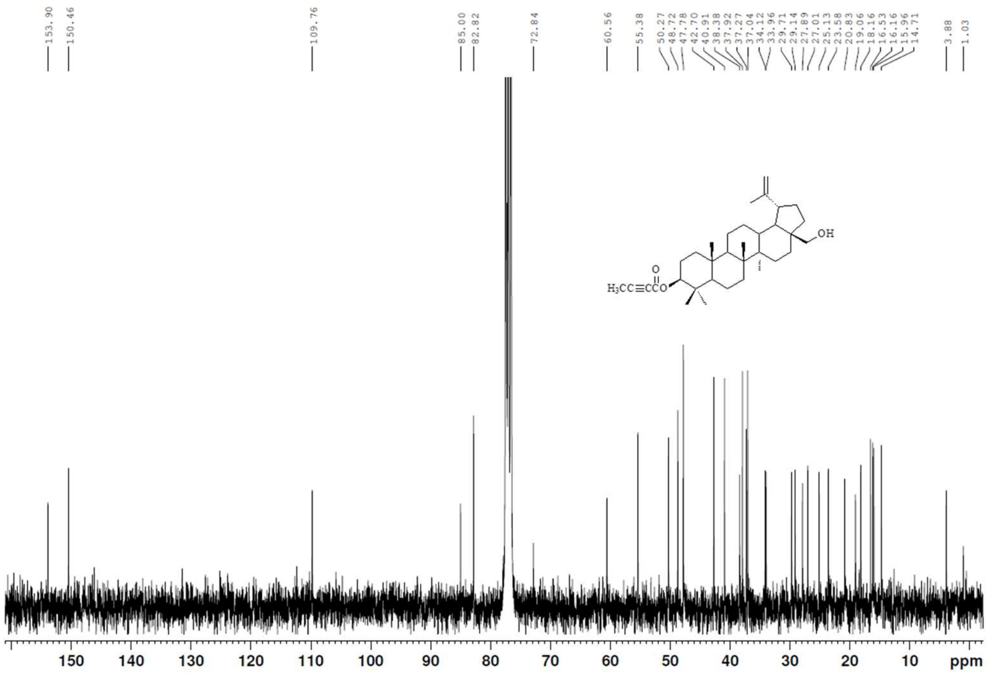

Figure S11: ^{1}H NMR spectrum of compound **10**

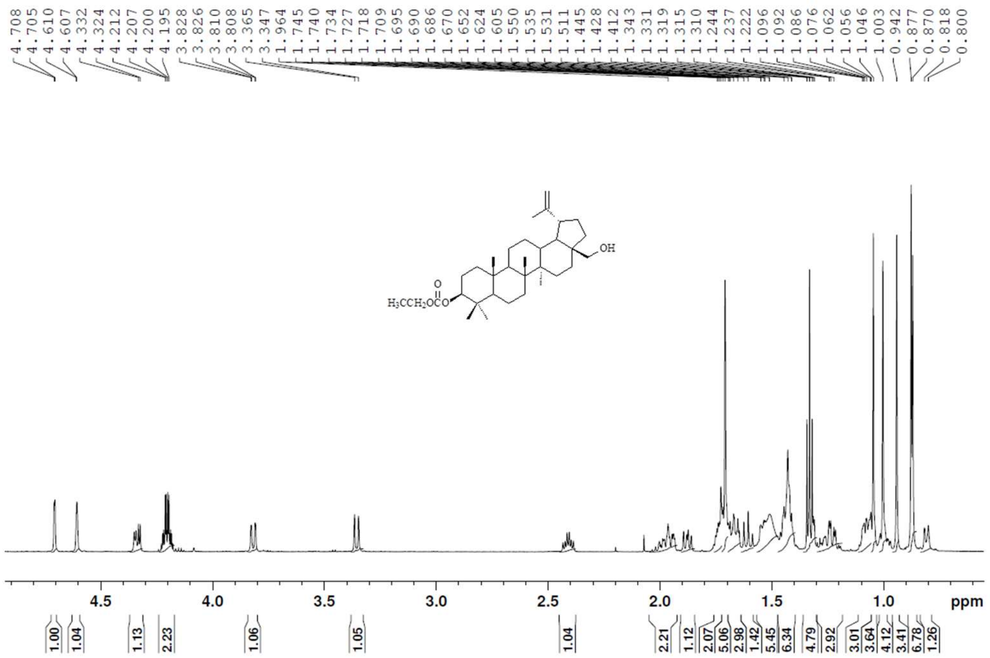

Figure S12: ^{13}C NMR spectrum of compound **10**

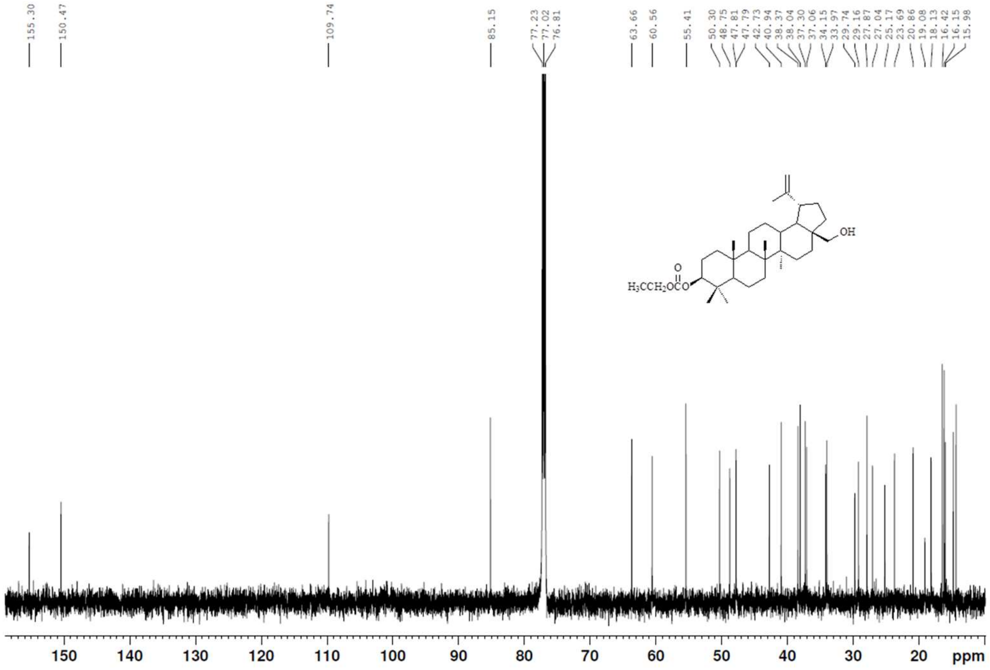

Figure S13: ^{1}H NMR spectrum of compound **11**

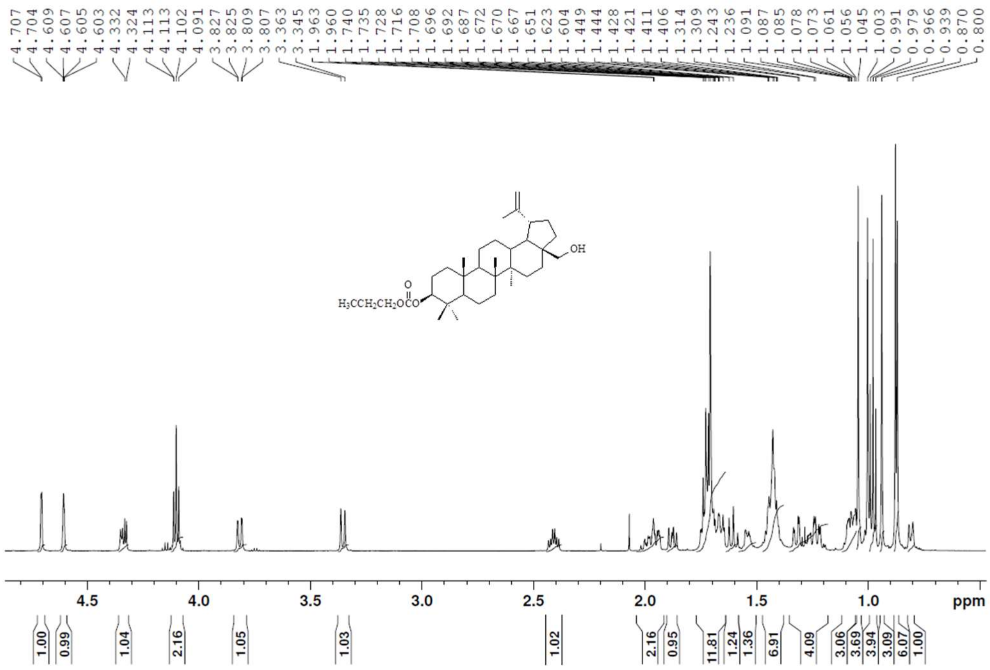

Figure S14: ^{13}C NMR spectrum of compound **11**

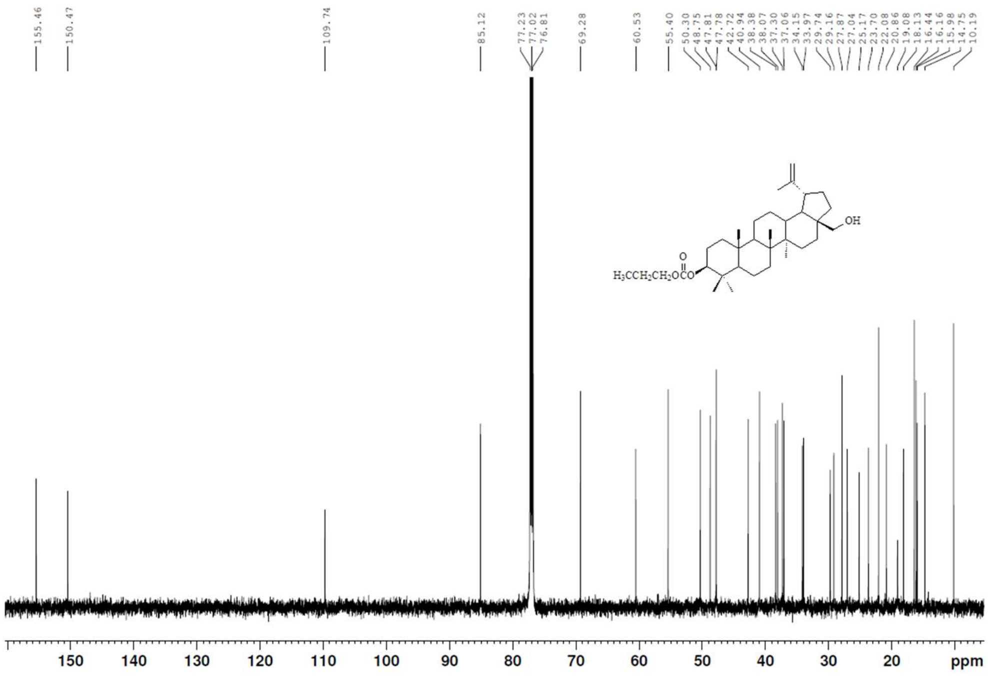

Figure S15: ^{1}H NMR spectrum of compound **12**

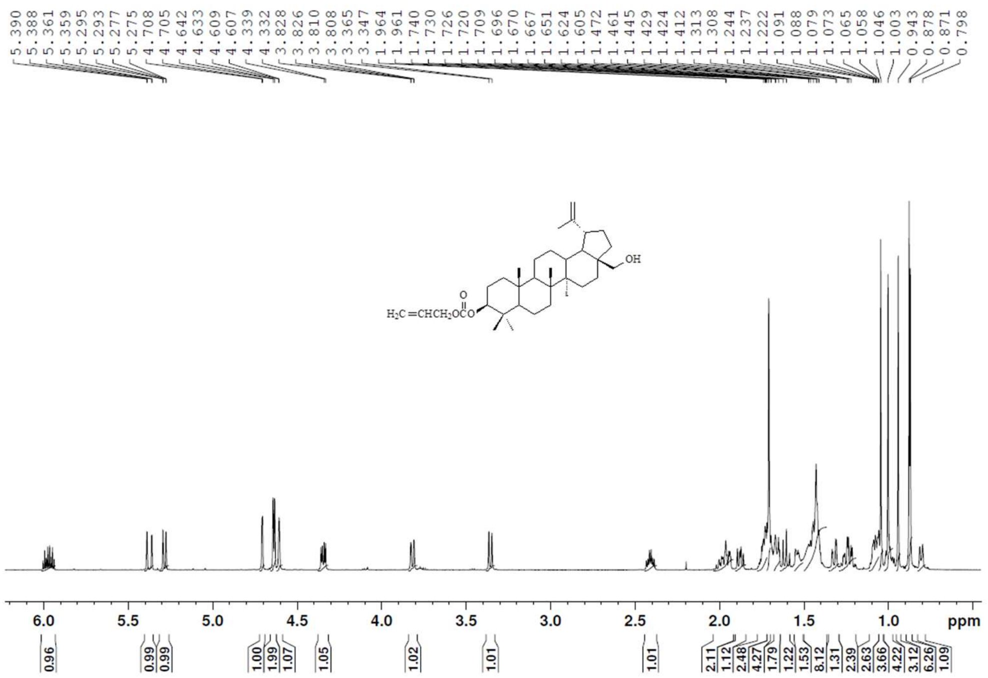

Figure S16: ^{13}C NMR spectrum of compound **12**

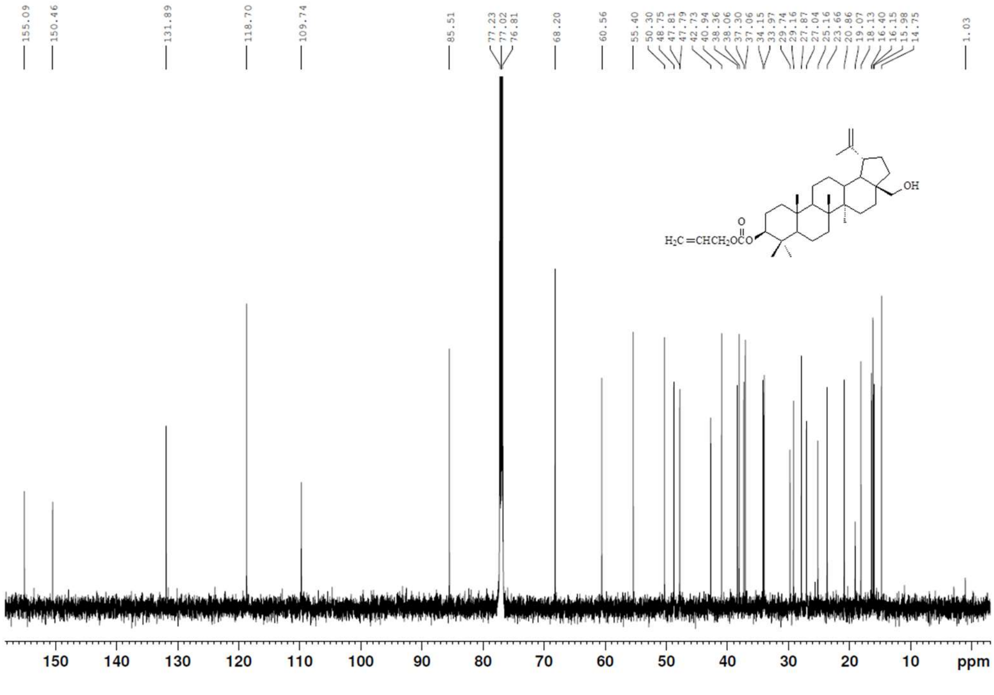

Figure S17: ^{1}H NMR spectrum of compound **13**

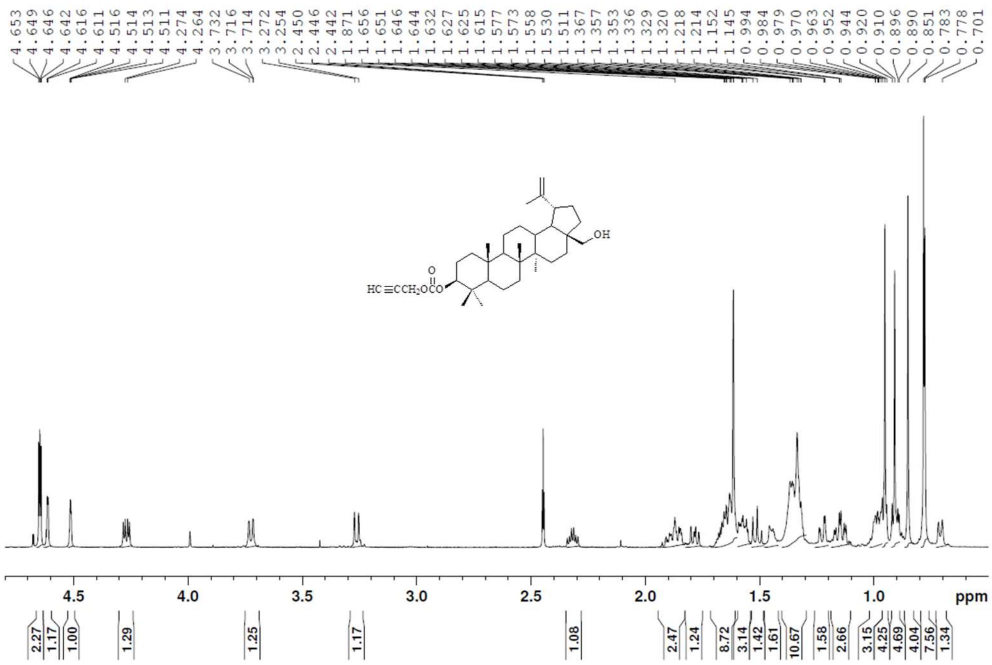

Figure S18: ^{13}C NMR spectrum of compound **13**

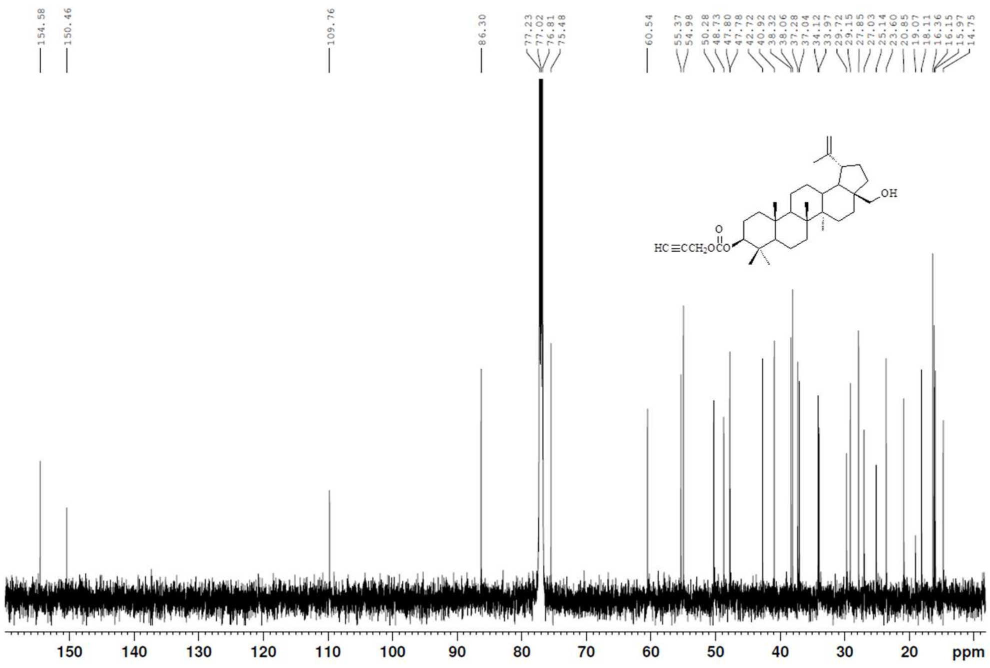

Figure S19: ^{1}H NMR spectrum of compound **14**

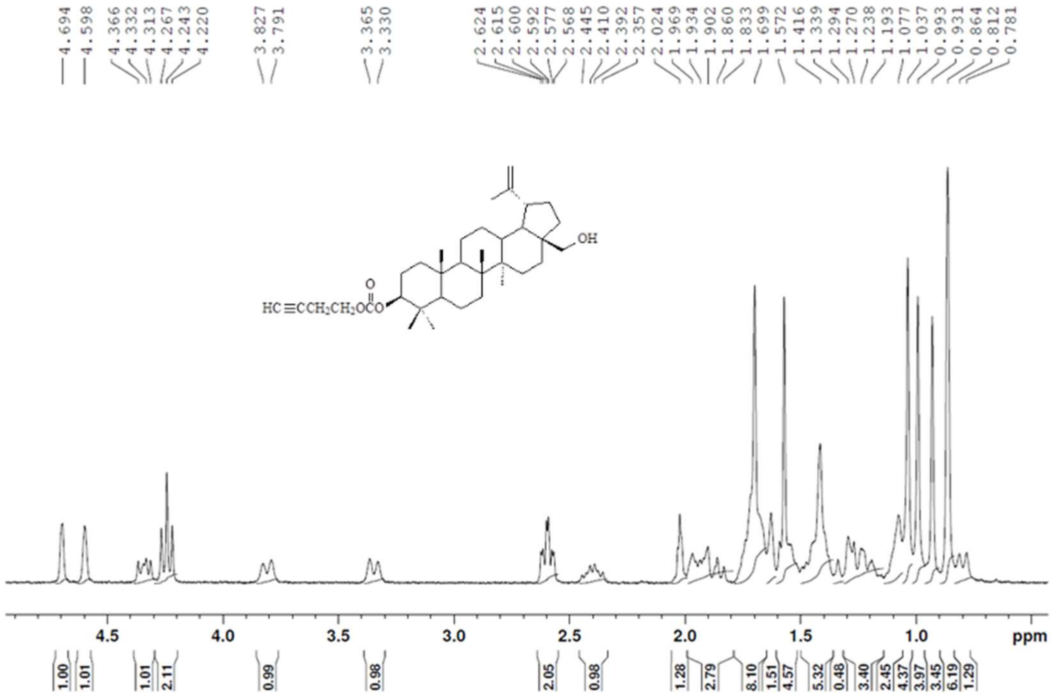

Figure S20: ^{13}C NMR spectrum of compound **14**

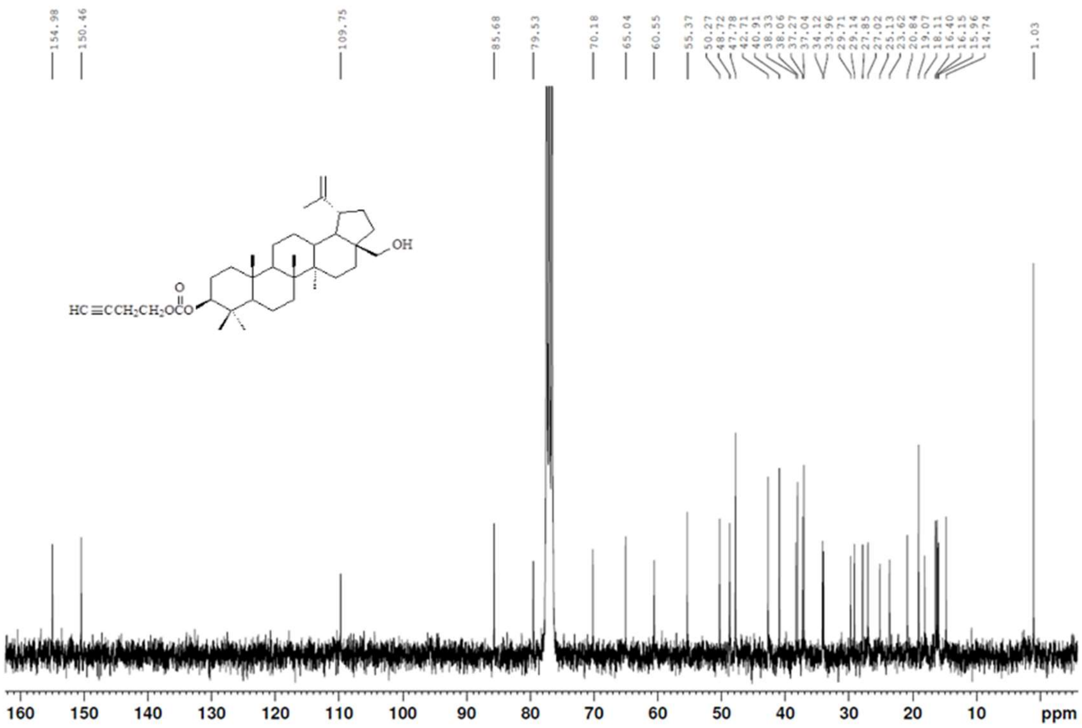

^{1}H NMR and ^{13}C NMR spectra of 3-modified betulinic aldehyde derivatives 1**5**-**24**

Figure 21: ^{1}H NMR spectrum of compound **15**

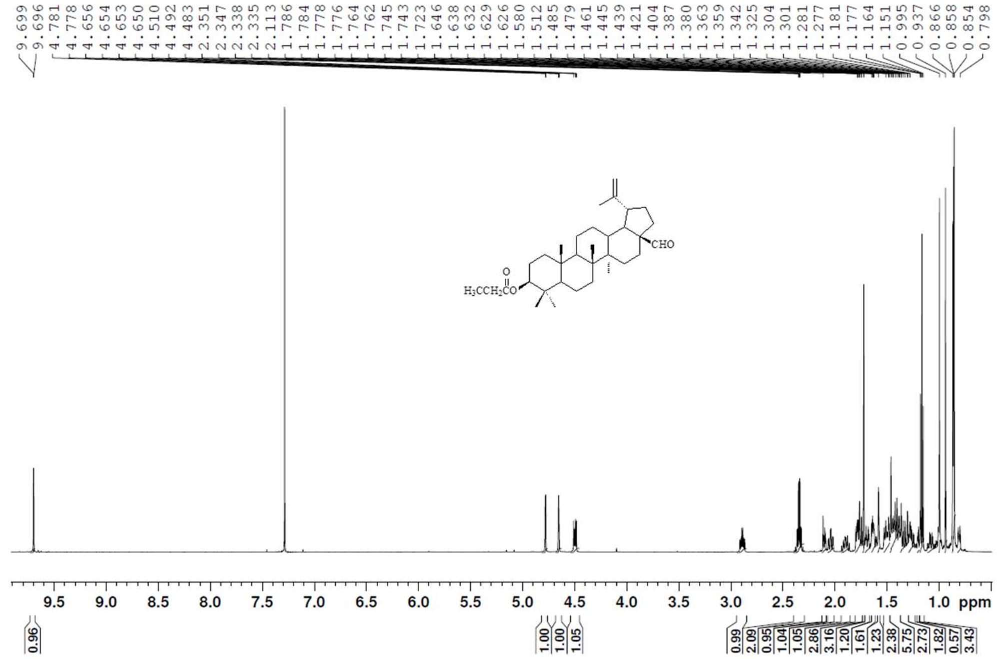

Figure 22: ^{13}C NMR spectrum of compound **15**

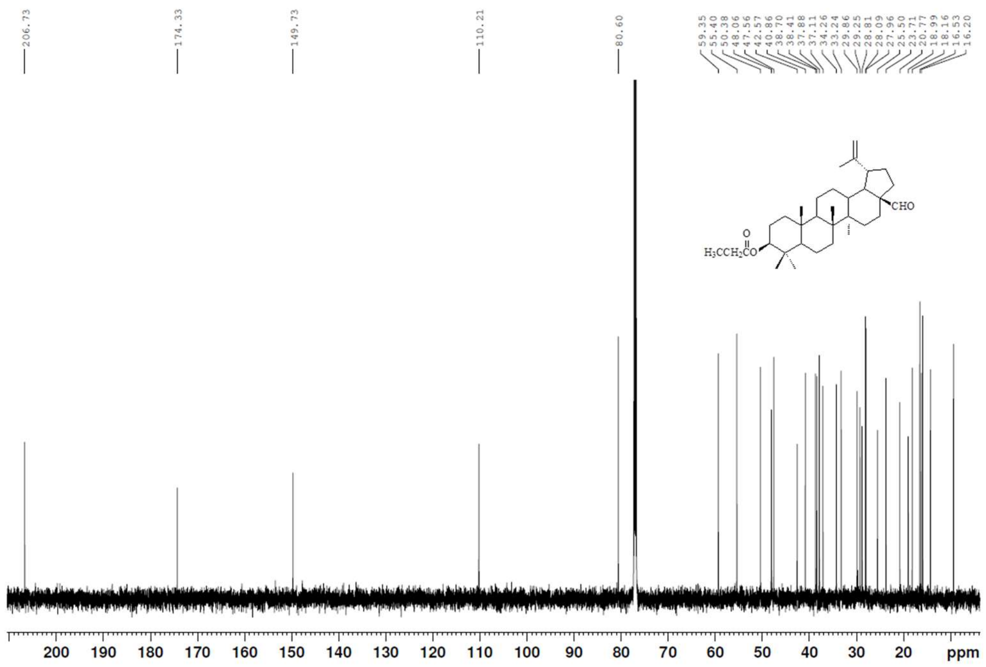

Figure 23: ^{1}H NMR spectrum of compound **16**

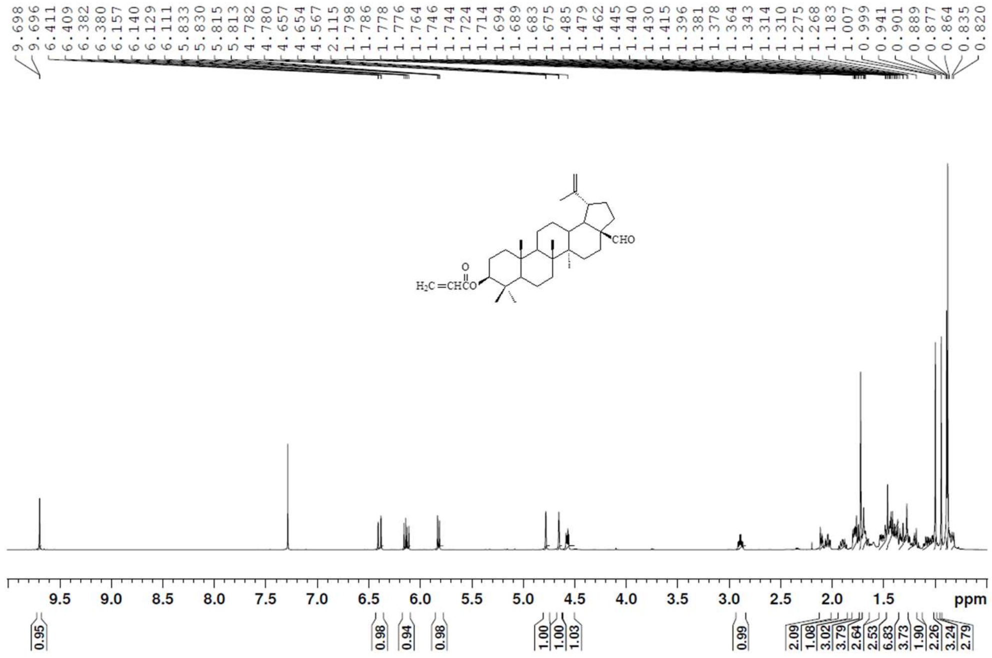

Figure 24: ^{13}C NMR spectrum of compound **16**

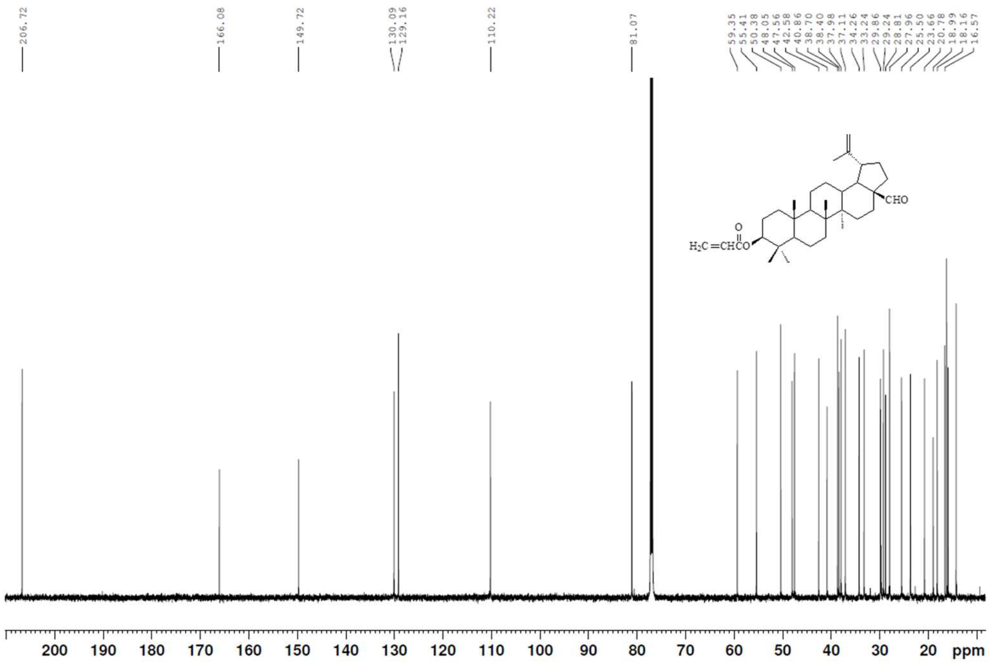

Figure 25: ^{1}H NMR spectrum of compound **17**

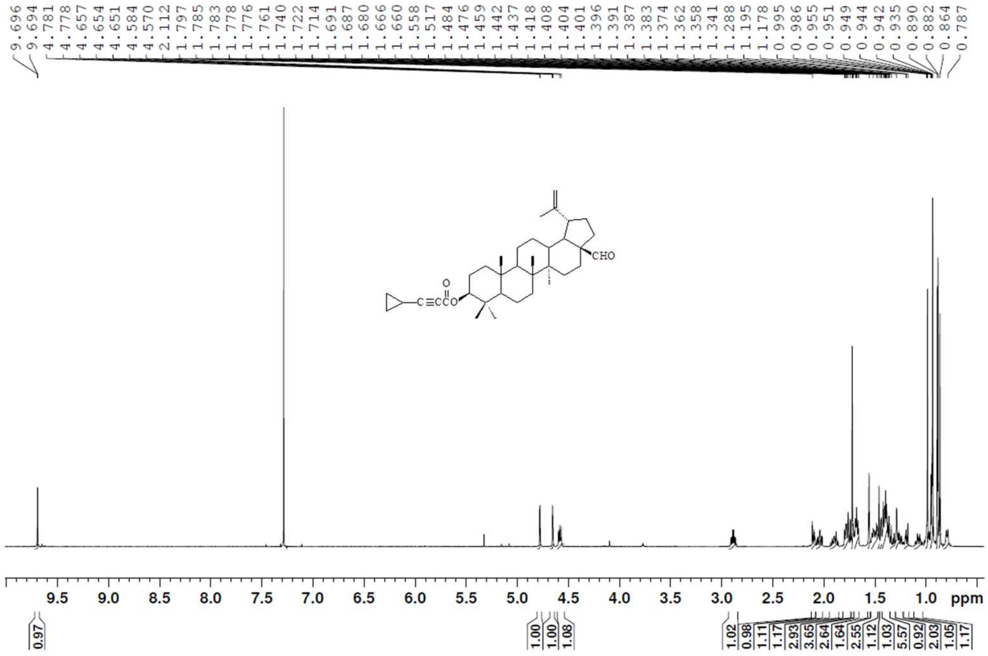

Figure 26: ^{13}C NMR spectrum of compound **17**

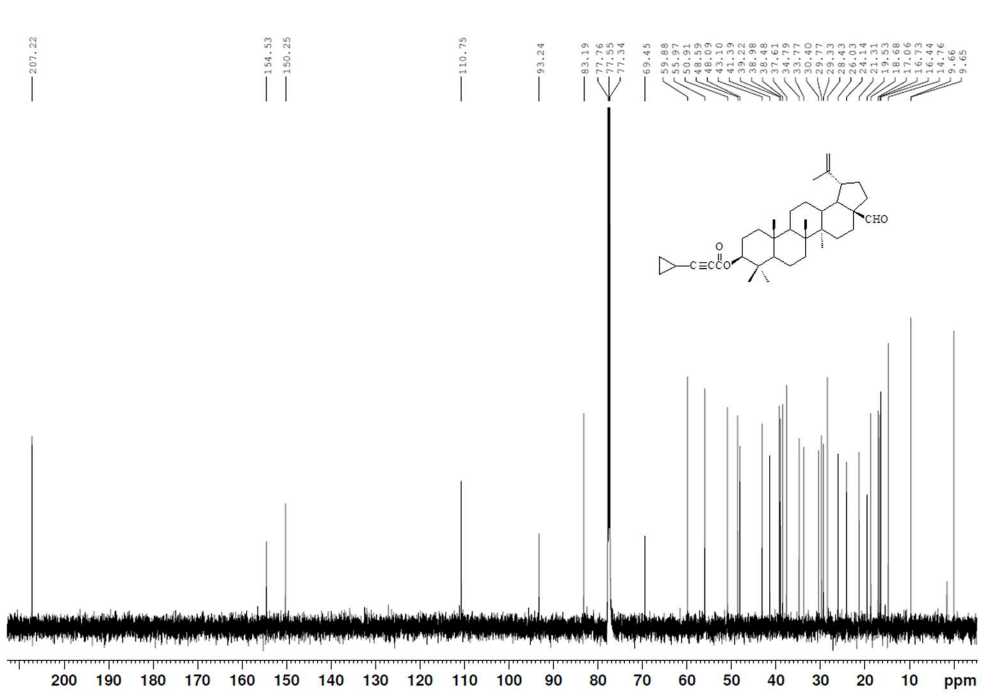

Figure 27: ^{1}H NMR spectrum of compound **18**

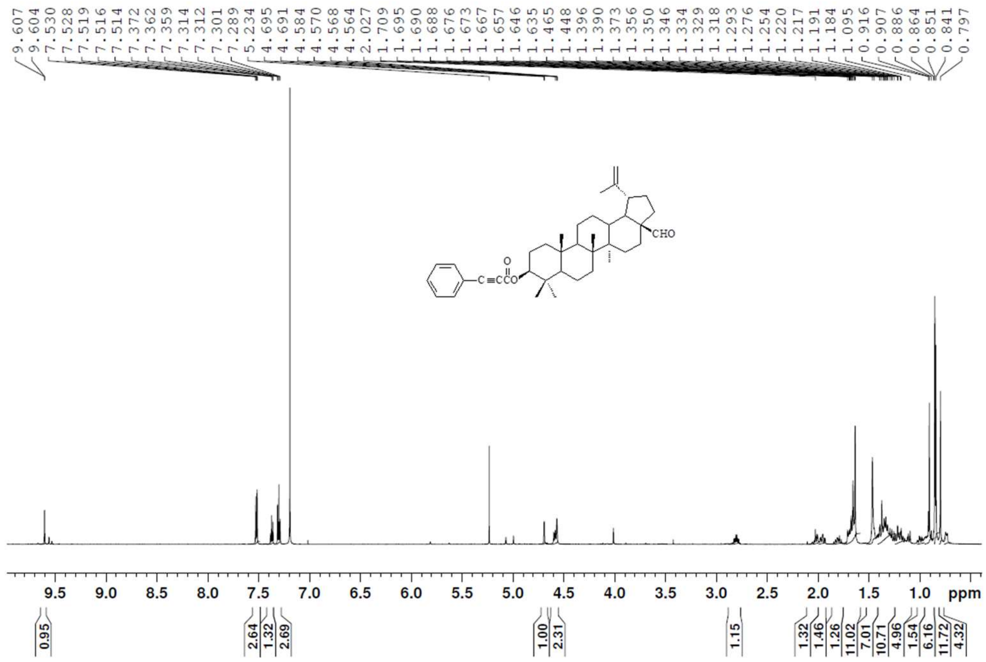

Figure 28: ^{13}C NMR spectrum of compound **18**

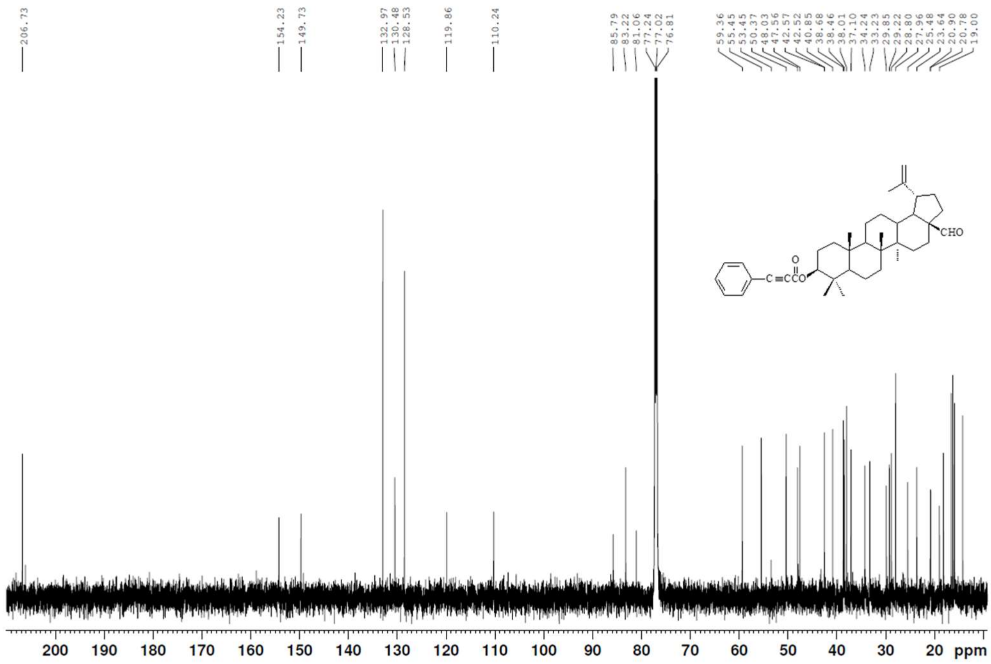

Figure 29: ^{1}H NMR spectrum of compound **19**

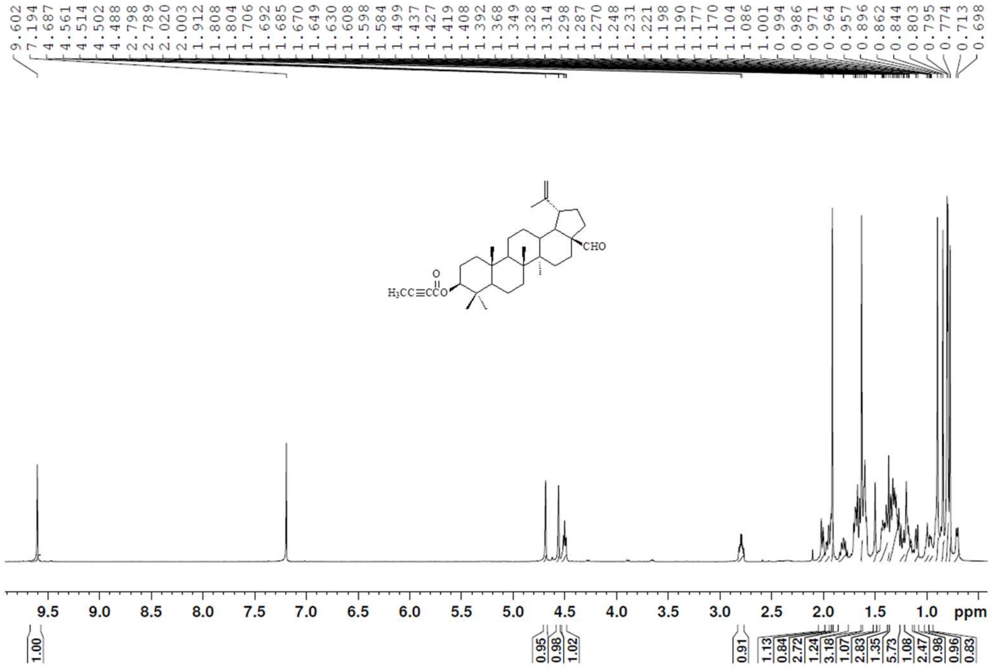

Figure 30: ^{13}C NMR spectrum of compound **19**

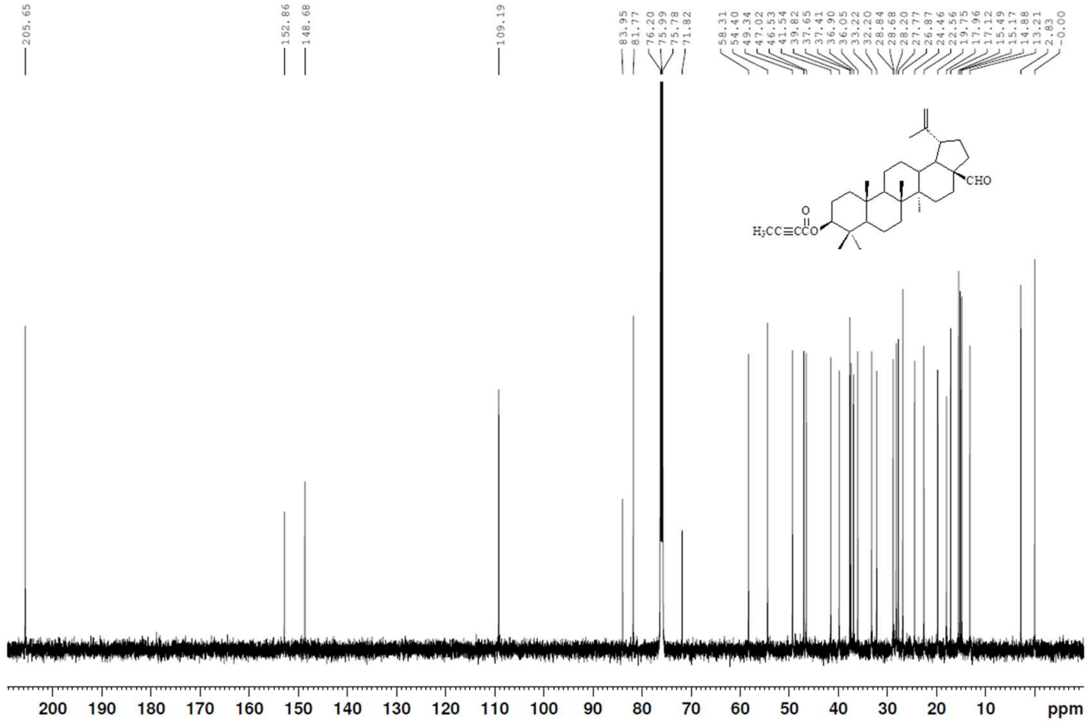

Figure 31: ^{1}H NMR spectrum of compound **20**

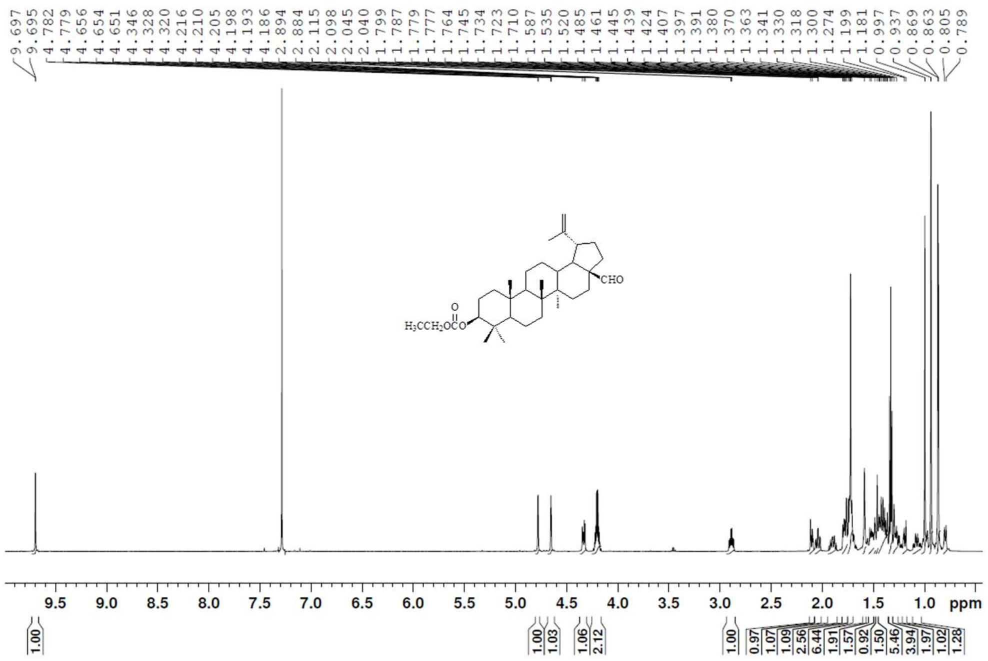

Figure 32: ^{13}C NMR spectrum of compound **20**

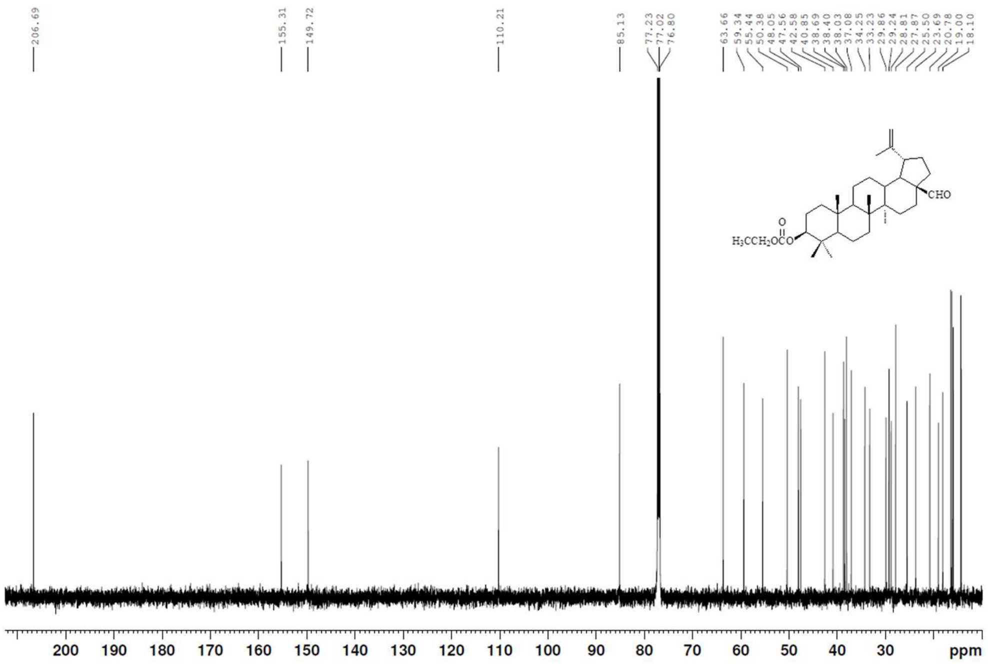

Figure 33: ^{1}H NMR spectrum of compound **21**

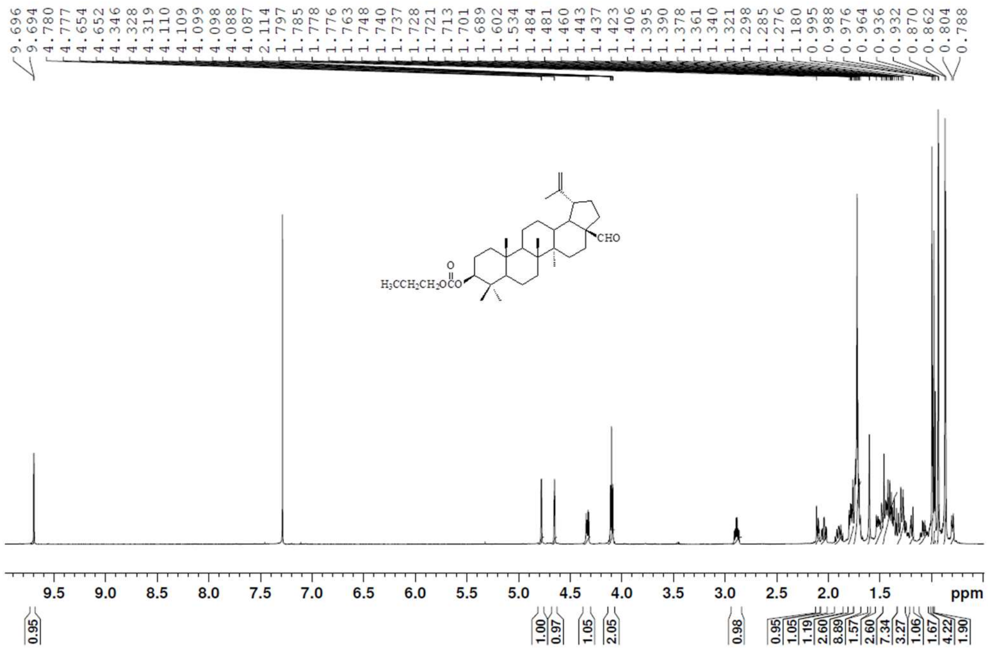

Figure 34: ^{13}C NMR spectrum of compound **21**

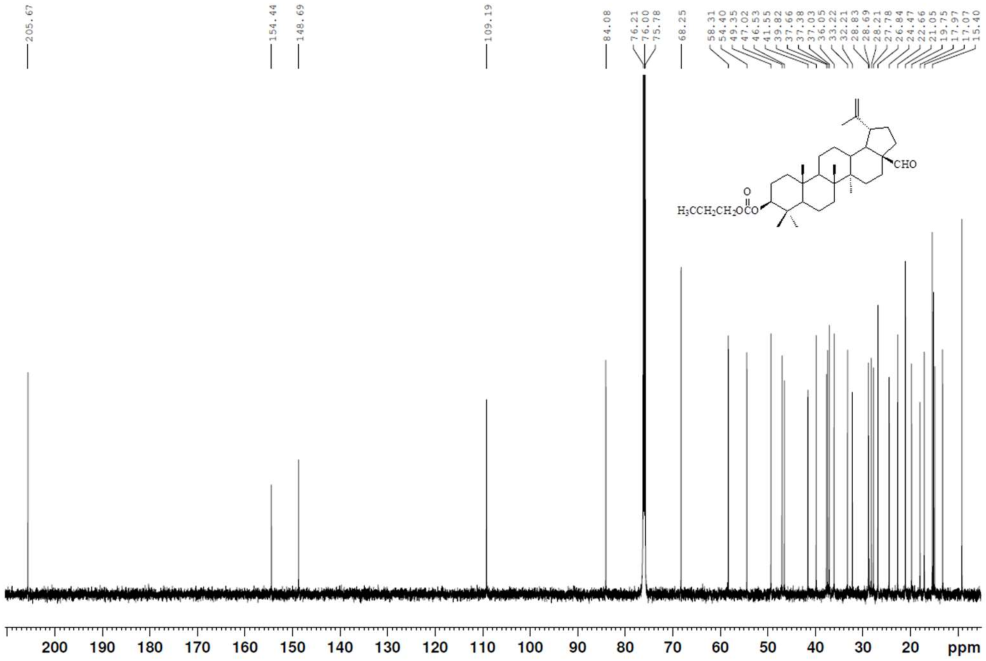

Figure 35: ^{1}H NMR spectrum of compound **22**

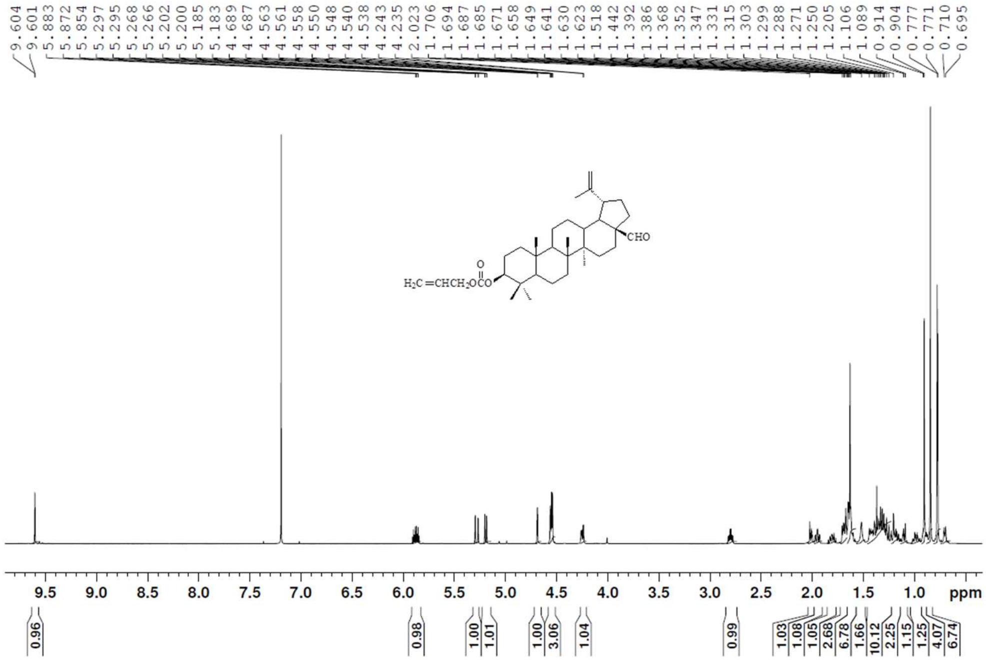

Figure 36: ^{13}C NMR spectrum of compound **22**

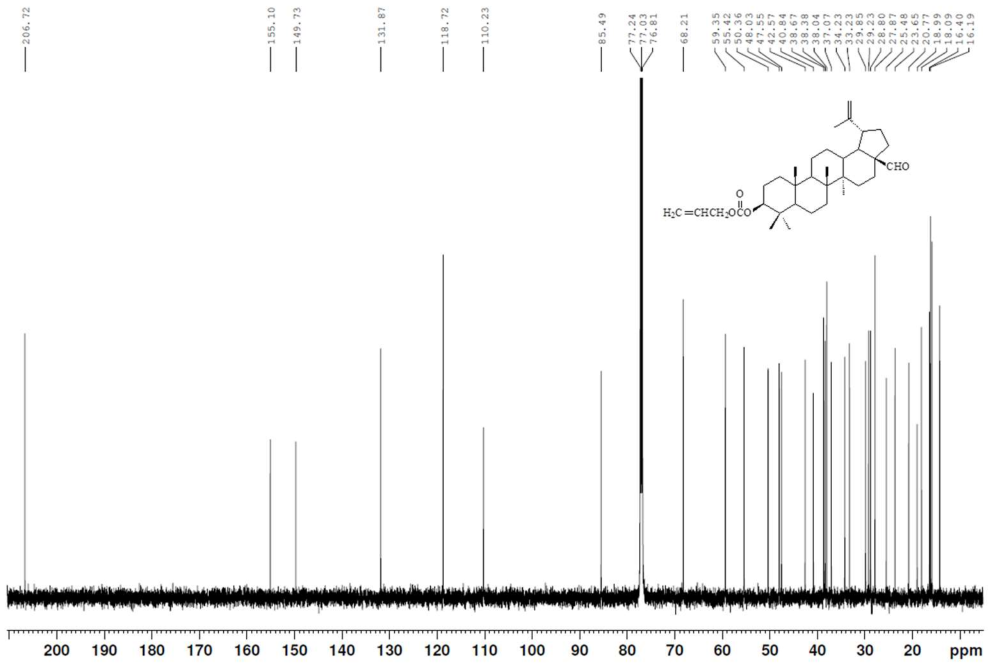

Figure 37: ^{1}H NMR spectrum of compound **23**

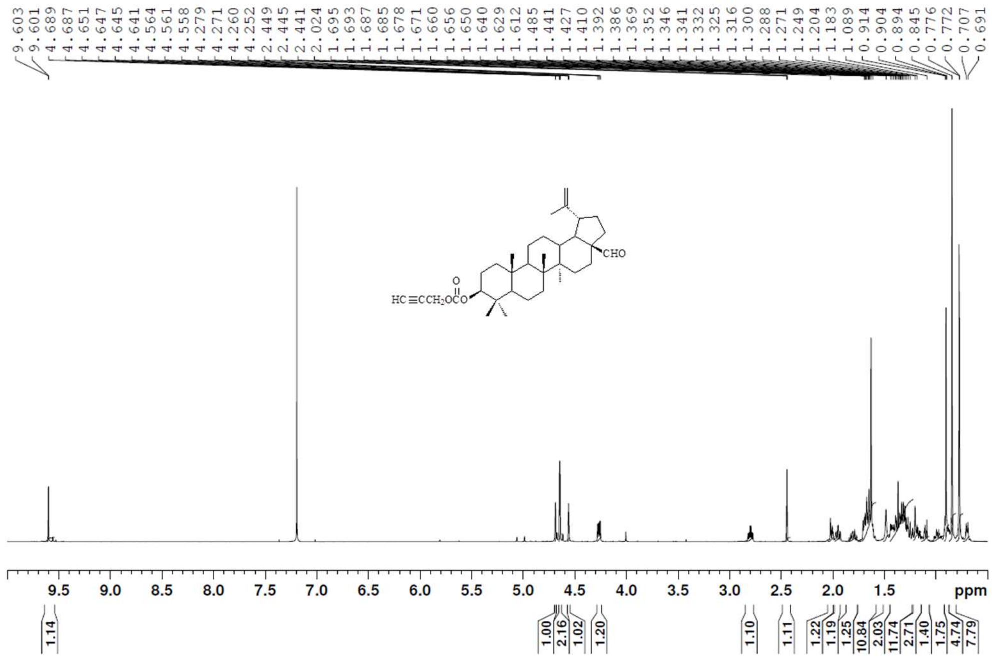

Figure 38: ^{13}C NMR spectrum of compound **23**

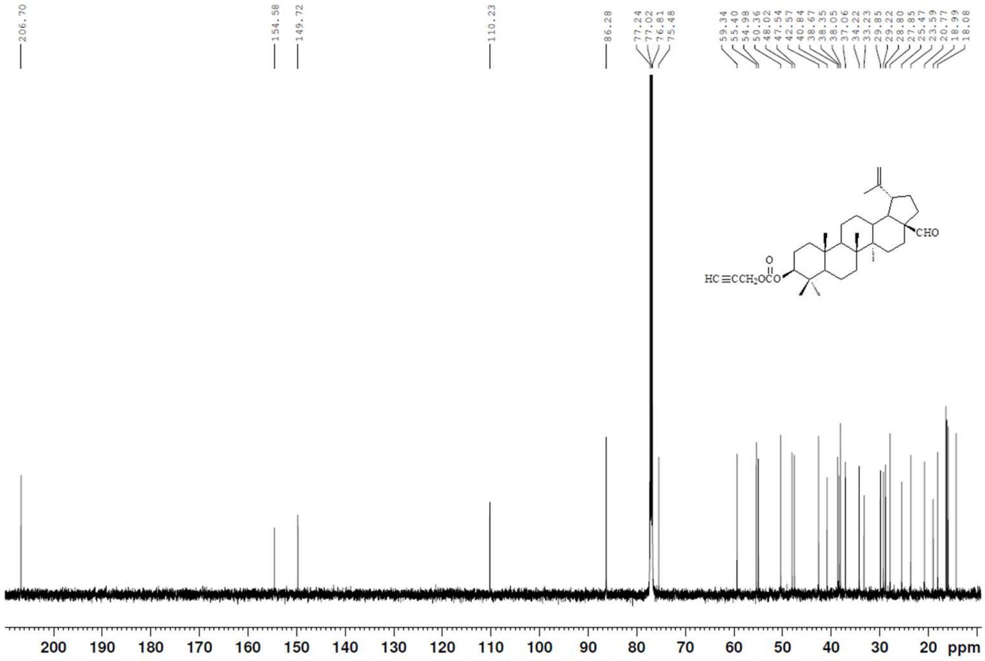

Figure 39: ^{1}H NMR spectrum of compound **24**

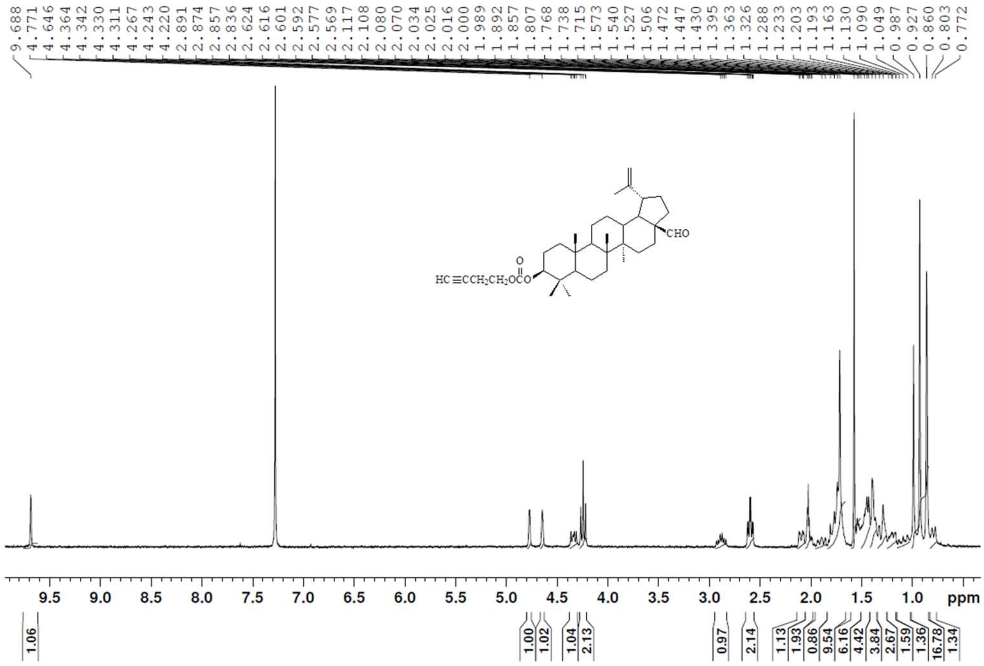

Figure 40: ^{13}C NMR spectrum of compound **24**

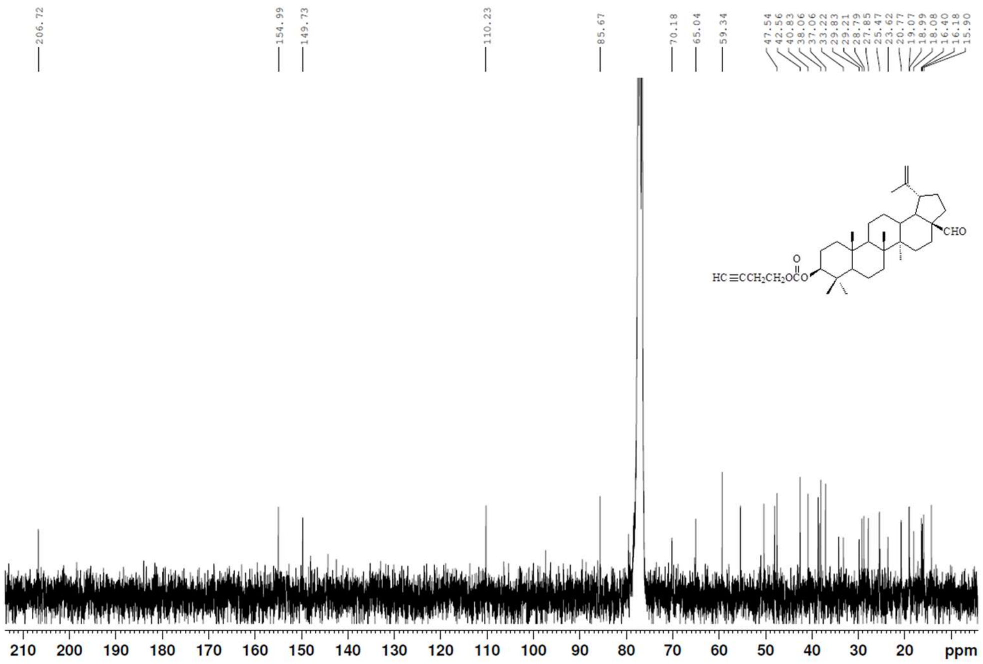
206.72
154.99
149.73
110.23
85.67
70.18
65.04
59.34
47.54
42.56
40.83
38.06
37.06
33.22
29.83
29.21
28.79
27.85
25.47
23.62
20.77
19.07
18.99
18.08
16.40
16.18
15.90
CHO
HC≡CCH2CH2OCO
O
210 200 190 180 170 160 150 140 130 120 110 100 90 80 70 60 50 40 30 20 ppm
